# Threat from peers: The effect of leaders’ voice endorsement on coworkers’ self-improvement motivation

**DOI:** 10.3389/fpsyg.2022.724130

**Published:** 2022-10-13

**Authors:** Pan Liu, Daiheng Li, Xiaoyan Zhang

**Affiliations:** ^1^Beijing Institute of Petrochemical Technology, Beijing, China; ^2^Beijing Academy of Safety Engineering and Technology, Beijing, China; ^3^Business School, Beijing Wuzi University, Beijing, China; ^4^School of Business, Beijing Technology and Business University, Beijing, China

**Keywords:** voice endorsement, perceived status threat, self-improvement motivation, trait competitiveness, self-evaluation maintenance theory

## Abstract

Existing studies mainly explore the antecedents of voice endorsement and its distal outcomes on voicers themselves. However, few have examined the mechanism of leaders’ voice endorsement on the voicers’ coworkers. Drawing on the self-evaluation maintenance theory, this paper uses perceived status threat as the mediator and trait competitiveness as the moderator to construct a conceptual model to explore the effect of leaders’ voice endorsement on coworkers’ self-improvement motivation. Through an empirical study with 279 sets of questionnaires from a manufacturing enterprise in China, the results show that leaders’ voice endorsement has a positive effect on coworkers’ self-improvement motivation through coworkers’ perceived status threat and that coworkers’ trait competitiveness can strengthen the positive relationship between leaders’ voice endorsement and coworkers’ perceived status threat. In addition, coworkers’ trait competitiveness can strengthen the indirect effect of leaders’ voice endorsement on coworkers’ self-improvement motivation *via* coworkers’ perceived status threat. The theoretical and practical implications of these findings are discussed.

## Introduction

Voice endorsement refers to the extent to which leaders give favorable valuations to the suggestions proposed by voicers (the employee with initiative who expresses opinions, concerns, or ideas to the leader about work-related issues; Van Dyne et al., 2003), and leaders’ willingness to implement the endorsed ideas into the practices (Burris, 2012). The ever-changing internal and external environment of organizations requires leaders to make more rapid and effective decisions to deal with potential risks and opportunities. In this context, employees’ voice behavior, as a key driver for improving decision quality and organizational effectiveness, has great significance for organizational development and change (Van Dyne and Lepine, 1998; Argote and Ingram, 2000). Leaders endorse constructive ideas that employees express can help them avoid or correct mistakes and improve work processes and outputs (Lam et al., 2019; He et al., 2020).

Due to the importance of voice behavior and voice endorsement in enhancing organizational running and effectiveness (Burris, 2012), existing studies have focused on the antecedents of voice endorsement, ranging from voicer factors (Whiting et al., 2012; Howell et al., 2015), leader factors (Fast et al., 2014; Li et al., 2019; Sherf et al., 2019), to voice strategies (e.g., Burris, 2012; Lam et al., 2019). Moreover, some literature (e.g., Corgnet and Hernán-González, 2014; Wei et al., 2015; Lam et al., 2018) has explored voicers’ responses to voice endorsement in the workplace. For example, research verified that voice endorsement makes voicers react favorably to their leaders (e.g., Burris, 2012; Burris et al., 2017; Lam et al., 2019), and voicers are more likely to engage in more subsequent positive behavior in the workplace (Janssen and Gao, 2015; Wei et al., 2015; Lam et al., 2018). In general, the focus of these studies is on how leaders’ voice endorsement benefits the voicers themselves.

Although the existing research has integrated the antecedent variables and explored some distal outcomes of voice endorsement, there is still relatively little research conducted on the effect of leaders’ voice endorsement on the voicers’ coworkers, and its outcomes and mechanisms have not yet been clarified. In the work team, employees’ voice behavior and leaders’ voice endorsement can have an impact not only on the voicers themselves but also on the coworkers around them. Milliken et al. (2003) have argued that due to the negative impact that voice may have on other employees, employees will consider social relationships and social factors when choosing to voice. Voice endorsement can show the voicers’ work ability, which helps them obtain approval and praise from the leaders, and thus improves their social status in the group (Morrison, 2011). Some scholars have pointed out that employees who perform well are often punished and ostracized because they make others look bad (Pleasant and Barclay, 2018). As a result, the voicers’ superior performance may make their coworkers feel jealous, which may result in anti-social punishments (e.g., workplace ostracism and workplace incivility) against the voicers (Wu et al., 2019).

**Figure 1:**
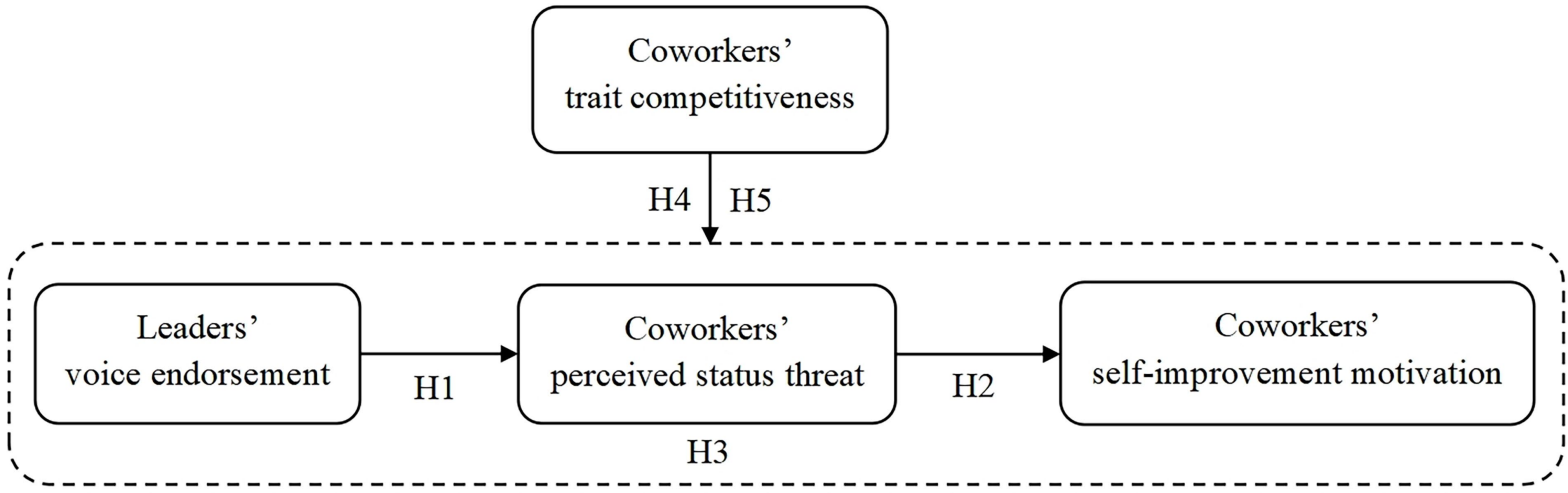
Conceptual model.

However, every coin has its two sides. Our study argues that although the outstanding performance of the voicers may make their coworkers feel status threatened, it may also cause coworkers to actively promote themselves to deal with the threat posed by the voicers. Self-evaluation maintenance theory is introduced as a theoretical framework to explore the mechanism between leaders’ voice endorsement and coworkers’ self-improvement motivation. The theory suggests that people are motivated to act from maintaining or enhancing their self-evaluation, and the relationship with others is a key factor that influences their self-evaluation (Zell and Alicke, 2010; Nicholls and Stukas, 2011). Employees whose suggestions are endorsed by leaders are generally regarded as more capable and reputable. In this situation, their coworkers may be slightly inferior when they are set off by excellent voicers, which makes coworkers have lower evaluations of themselves and perceive threats to their status. To maintain or improve their self-evaluation, coworkers have to take steps to improve their performance in response to potential status threats. In addition, whether the personality traits affect the outcome of the voice endorsement is also a concern of our study. This study introduces trait competitiveness as a moderating factor to investigate the impact of leaders’ voice endorsement on coworkers’ self-improvement motivation. The conceptual model is depicted in Figure 1.

Our research advances the extant literature in multiple ways. First, this study shifts attention from the antecedents of voice endorsement to its distal outcomes of coworkers. Our research contributes to the voice literature by exploring whether, how, and under what conditions leaders’ voice endorsement impacts coworkers’ self-improvement motivation. It also responds to (Morrison’s 2011) suggestion that future research should focus on the reactions of the voicers’ coworkers to the voice. Second, this study advances the research on the perceived status threat by revealing the positive impact of leaders’ voice endorsement on coworkers. This provides a new pathway for the current research on the perceived status threat that mostly focuses on its negative effects. Third, we contribute to the understanding of when coworkers are more likely to possess self-improvement motivation after the leaders’ voice endorsement by highlighting the importance of coworkers’ trait competitiveness. Finally, we extend the self-evaluation maintenance theory by illustrating the coworkers’ psychological responses to leaders’ voice endorsement. From the viewpoint of self-evaluation and self-improvement, our research provides a new theoretical perspective and explanation for how leaders’ voice endorsement impacts coworkers’ self-improvement motivation *via* coworkers’ perceived status threat.

## Theory and hypotheses

### Voice endorsement and perceived status threat

Voice endorsement is generally considered as the leaders’ positive evaluation of employees’ voice and they are willing to implement the endorsed ideas in practice (Burris, 2012). It can increase the popularity of voicers, leading to good performance evaluations and promotion opportunities (Lam et al., 2019). Weiss and Morrison (2019) have found that voice endorsement can enhance the status of the person giving the advice in the team. Status is defined as the respect and appreciation that an individual receives from others based on the perceived social value of himself/herself (Anderson et al., 2015). In organizations, competition for status can be intense and persistent (Bendersky and Hays, 2012). The status holders often face threats and challenges from other coworkers. Perceived status threat means that individuals perceive that their status is challenged by others and may lead to potential loss of status (Kellogg, 2012). This perception may prompt individuals to take action to protect their current threatened status.

This study argues that leaders’ voice endorsement may make their coworkers perceive status threats. First, the self-evaluation maintenance theory points out that when those around the individual are prominent, the individual’s self-evaluation decreases, resulting in a range of worries and tensions (Zell and Alicke, 2010). Morrison and Milliken (2000) have found that employees’ voices can help leaders make effective decisions and identify problems and risks. As a result, leaders tend to have higher evaluations of the voicers’ ability and performance after they endorse the high-quality voice (Whiting et al., 2008). The suggestions of voicers can help the organization progress and achieve success. Their efforts and contributions can be recognized and accepted by leaders, and they will be awarded high status (Ridgeway, 1982). The voicers’ elevated status and outstanding performance can overshadow their coworkers, making them perceive that they are in danger of being replaced. Second, leaders’ voice endorsement may touch upon vested interests, which, in turn, may make them feel threatened. Employees’ voice points out the problems in the organization. They hope that the organizational situation can change for the better (Van Dyne and Lepine, 1998). However, leaders’ voice endorsement may have implications for the interests of those who have a vested interest in the organization (e.g., coworkers). Given that status depends on the granting of others, employees’ voice behavior and leaders’ voice endorsement may change the internal systems of the organization by touching on resources that coworkers already have, the coworkers may feel that their status is threatened (Hays and Blader, 2017). To summarize the above, this study presents the following hypothesis:

*Hypothesis 1*: Leaders’ voice endorsement has a positive effect on coworkers’ perceived status threat.

### Perceived status threat and self-improvement motivation

Self-improvement motivation refers to an individual’s tendency to spontaneously or motivatedly improve a particular ability (Halliwell and Dittmar, 2005). It usually prompts people to make upward comparisons with goals that are believed to inspire specific personalities. Festinger (1954) believes that people constantly evaluate themselves to clarify their status. In the absence of norms and standard objective information, individuals make subjective comparisons with others around them to obtain an accurate self-evaluation. Subjective comparisons can occur even when objective information is available. Individuals not only make self-evaluations but also make social comparisons for self-improvement (Marsh and Parker, 1984). Self-improvement motivation is the result of individual subjective social comparison.

In organizations, status, as a scarce social resource, can bring many benefits to the status holder, such as more chances, a greater influence on others, and priority over resources (Simonton, 2003). As a result, people are eager to upgrade their status or maintain their current status (Anderson et al., 2015). The specificity of status also makes inevitable status competition between coworkers. According to the self-evaluation maintenance theory, comparisons are more pronounced when the relevance of the two parties is high. The outstanding performance of the voicers may dwarf their coworkers, which reduces the self-evaluation of coworkers (Zell and Alicke, 2010). The perceived status threat is the reaction of coworkers after comparing themselves with the behavior and performance of the voicer, which implies a decrease in the coworkers’ self-evaluation. When a status threat is perceived, coworkers respond by taking steps to maintain or improve their self-evaluation. For example, coworkers who perceive status threats may try to reduce the performance of the voicers or try to improve themselves. Since “bad-mouthing” to the voicers may bring risks to their reputation and image, coworkers may be more inclined to take less risky measures, that is, strive to improve their capabilities to maintain or enhance their status in the organization (Nicholls and Stukas, 2011). In addition, according to the self-evaluation maintenance theory, aggressive behavior against the voicers that potentially damages coworkers’ image does not improve the evaluations of coworkers themselves. Only by developing and improving their capabilities can coworkers truly maintain their positions and establish good self-evaluation. Therefore, when faced with status threats, internal self-improvement by coworkers is their highest priority option. The above discussion is summarized in the following hypothesis:

*Hypothesis 2*: Coworkers’ perceived status threat has a positive effect on their self-improvement motivation.

Based on this, our research further proposes that the coworkers’ perceived status threat plays a mediating role in the relationship between leaders’ voice endorsement and coworkers’ self-improvement motivation. While leaders’ voice endorsement enhances voicers’ status and image, the changes advocated by the voicers may also threaten the vested interests and status of coworkers. To maintain their position, coworkers will actively take measures to deal with such threats. The risks brought by direct interference with the voicers make coworkers more likely to find breakthroughs from internal causes, that is, to use self-improvement to improve their self-evaluation and maintain their status (Halliwell and Dittmar, 2005). To summarize the above, this study presents the following hypothesis:

*Hypothesis 3*: Coworkers’ perceived status threat plays a mediating role in the relationship between leaders’ voice endorsement and coworkers’ self-improvement motivation.

### The moderating effect of coworkers’ trait competitiveness

Individual behavior is determined by the interaction of individual and situational factors, and individual traits can influence how they construct and react to a given situation (Schneider, 1983). Some studies have shown that individuals’ responses to the same source of stress are different due to the differences in their personality traits, which can inhibit or amplify their responses to stress or threat (Dandeneau et al., 2007; Grant and Langan-Fox, 2007; Sadiković et al., 2020). Therefore, this study suggests that differences in the level of trait competitiveness may cause coworkers to react differently when they perceive the status threat.

Trait competitiveness is a personality trait in which individuals enjoy interpersonal competition and desire to win and be better than others (Ferguson, 1984). Existing research has indicated that competitiveness can act as a positive trait that motivates individuals to achieve their goals. However, it can also have negative effects. For example, it can drive individuals to win at all costs and may even produce criminal or interpersonal conflict behaviors (Jelinek and Ahearne, 2010). In addition, compared with people with low trait competitiveness, people with high trait competitiveness are more likely to develop a differentiated mentality that opposes others (Babalola et al., 2022).

These characteristics of trait competitiveness indicate that individuals with high trait competitiveness may be more sensitive to their status gains and losses. In the organization, due to the individuals’ competitive mentality, they are more likely to view coworkers as competitors for the organizational resources and opportunities (e.g., bonuses, promotions) rather than teammates who complete tasks and goals together. When confronted with outstanding voicers whose suggestions are endorsed by leaders, they feel more competitive pressure and worry about whether their position will be replaced. Thus, coworkers with high trait competitiveness may react more strongly to the outstanding performance of the voicers and care more about the gain or loss of their status. The above discussion is summarized in the following hypothesis:

*Hypothesis 4*: Coworkers’ trait competitiveness moderates the relationship between leaders’ voice endorsement and coworkers’ perceived status threat such that this positive relationship is stronger when coworkers’ trait competitiveness is higher rather than lower.

As mentioned above, coworkers’ perceived status threat mediates the relationship between leaders’ voice endorsement and coworkers’ self-improvement motivation, and coworkers’ trait competitiveness moderates the relationship between leaders’ voice endorsement and coworkers’ perceived status threat. Competitive employees tend to actively strive for victory and motivate themselves to achieve their goals (Jelinek and Ahearne, 2010). Because trait competitiveness is associated with a differentiated mentality, employees with high trait competitiveness are more likely to evaluate voicers whose suggestions are endorsed by leaders as negative and to exaggerate the negative effects of competitors on them (Babalola et al., 2022). When perceiving status threats, they are more inclined to face the competition and improve themselves to gain greater advantage and status. To summarize the above, this study presents the following hypothesis:

*Hypothesis 5*: Leaders’ voice endorsement is related to coworkers’ self-improvement motivation via conditional indirect effect such that this positive relationship is mediated by coworkers’ perceived status threat and moderated by coworkers’ trait competitiveness.

## Materials and methods

### Sample and procedure

In this study, sample data were collected at a manufacturing company in Shanghai, China to test the hypothesized model. The company is mainly engaged in the production of elevator components, motors, inverters, servo drives, wires, and cables. China is the manufacturing center of the world, and Shanghai is the gathering area of Chinese manufacturing. This company is an important member of China’s most representative industrial manufacturing industry. We chose this company for the following reasons. This company brings together people from all over the country. They come from different and diversified backgrounds, which can truly reflect the current general situation of corporate personnel in China. Moreover, through interviews with employees, and discussions with company leaders, we found that voice behavior and voice endorsement was universal in this company. Most employees also believed that the leaders’ voice endorsement of other employees had an impact on their future work behavior. Given this situation, we believed that this company was appropriate to conduct our surveys.

To start the data collection process, the human resources department introduced the survey information to the employees, requested voluntary participation, and randomly selected all employees. After obtaining permission from the CEO of the company, we used a four-digit code to identify each participant. All participants were assured that their responses would remain confidential, and only be used for research purposes. After completing the questionnaires, the participants put them in sealed envelopes and handed the envelopes to the researchers. Each participant received a bonus (20RMB, 3USD) when completing all waves of the survey.

We targeted 469 pairs of employees and leaders so that the employee reported to their immediate supervisor. The purpose is to capture variance in different impacts of leaders’ voice endorsement on employees, because different supervisors may have varying levels of impact on employees. We used a matched four-digit code to identify each leader (e.g., 1001) and employee (e.g., 2001). The participants comprise 469 employees and 136 of their immediate leaders. To minimize common method bias, this study used a three-wave approach for data collection, with 1 month between each wave (Podsakoff et al., 2003). In the first wave, we distributed 136 questionnaires to leaders, with 120 valid questionnaires (88.2% return rate), and collected data on voice endorsement. In the second wave, we distributed 423 questionnaires to these leaders’ employees, with 358 valid questionnaires (84.6% return rate), and collected data on the perceived status threat, trait competitiveness, and control variables. In the third wave, we distributed 358 questionnaires to employees who had submitted valid questionnaires in wave two, with 295 valid questionnaires (82.4% return rate), and collected data on self-improvement motivation.

After eliminating the 16 invalid questionnaires (such as the same answers to all items or with missing values), the survey finally obtained 279 sets of valid questionnaires (59.5% effective rate). The basic characteristics of the employee sample are as follows. In terms of gender, males accounted for 51.6% and females accounted for 48.4%. In terms of age, 31–40 years old accounted for the largest proportion, about 25.8%. In terms of tenure, 1–5 years accounted for the largest proportion, about 40.5%. In terms of education, bachelor accounted for the largest proportion, about 34.1%.

### Measures

Since all the measures were originally constructed in English, we used the back-translation method to translate all the items. We invited two doctoral students to each independently translate the English items into Chinese, and then two bilingual experts in the field of organizational behavior translated the Chinese items back into English. Finally, all authors read and compared the original English items and the translated English items, to ensure accuracy in the translation. All variables were measured using a 7-point Likert-type scale (1 = “completely disagree” to 7 = “completely agree”). Appendix A shows the measurement scales in the present study.

#### Voice endorsement

We used (Burris’s 2012) 5-item scale to measure voice endorsement. A sample item is, “I think this employee’s comments should be implemented” (Cronbach’s *α* = 0.94).

#### Perceived status threat

The perceived status threat was assessed using the 4-item scale from Bendersky and Hays (2012). A sample item is, “My team members competed for influence” (Cronbach’s *α* = 0.91).

#### Self-improvement motivation

We used a 7-item scale from Breines and Chen (2012) to assess self-improvement motivation. A sample item is, “I want to find opportunities that will challenge me and help me grow as a good employee” (Cronbach’s *α* = 0.96).

#### Trait competitiveness

A 5-item scale developed by Wagner (1995) was used to measure trait competitiveness. A sample item is, “I believe that success is the most important thing in life” (Cronbach’s *α* = 0.94).

#### Control variables

Following the previous research on voice endorsement (Burris, 2012; Wei et al., 2015; Lam et al., 2019), we controlled for employees’ gender, age, tenure, and education to rule out the alternative explanations that those demographics influence the outcomes of interest.

## Results

### Confirmatory factor analysis

Confirmatory factor analysis (CFA) was conducted with Mplus 8.0. As shown in Table 1, all factor loadings exceeded 0.6 and were significant, suggesting that the item validity of measures was acceptable. The composite reliability (CR) of each construct was larger than 0.7, which suggested that composite reliability was acceptable. And the average variance extracted (AVE) by each construct is larger than 0.5, which illustrated that convergence validity was acceptable. The discriminate validity value (square root of AVE) of each construct was larger than the Pearson’s correlation value. Accordingly, all measures appear to exhibit acceptable values and validity.

**Table 1:** Results of confirmatory factor analysis of each measure.

Variables	Estimate	CR	AVE	1	2	3	4
Leaders’ voice endorsement	0.786–0.899	0.938	0.717	***0.847***			
Coworkers’ perceived status threat	0.810–0.890	0.909	0.714	0.610	***0.845***		
Coworkers’ self-improvement motivation	0.783–0.988	0.961	0.781	0.640	0.629	***0.884***	
Coworkers’ trait competitiveness	0.830–0.948	0.942	0.764	0.686	0.471	0.577	***0.874***

CR, composite reliability; AVE, average variance extracted; discriminate validity value of each construct is shown along the diagonal in bold italics.

### Descriptive analyses

Table 2 shows the means, standard deviations, and correlations among the variables in the present study. As shown in Table 2, voice endorsement may be related to perceived status threat (*r* = 0.61, *p* < 0.01) and self-improvement motivation (*r* = 64, *p* < 0.01), and the perceived status threat may be related to self-improvement motivation (*r* = 63, *p* < 0.01). These results provide the basis for subsequent hypotheses testing.

**Table 2:** Means, standard deviations, and correlations of the variables.

Variables	1	2	3	4	5	6	7	8
Age	1							
Gender	0.136^*^	1						
Tenure	0.379^**^	0.040	1					
Education	−0.502^**^	−0.166^**^	−0.306^**^	1				
Leaders’ voice endorsement	0.000	−0.010	0.065	0.077	***0.938***			
Coworkers’ perceived status threat	0.056	0.036	0.088	−0.022	0.610^**^	***0.908***		
Coworkers’ self-improvement motivation	0.043	−0.018	0.063	0.033	0.640^**^	0.629^**^	***0.966***	
Coworkers’ trait competitiveness	0.076	0.050	0.103	−0.023	0.686^**^	0.471^**^	0.577^**^	***0.940***
Mean	3.036	0.645	3.039	2.677	5.011	5.302	5.199	4.988
SD	1.354	0.479	1.282	0.995	1.233	1.240	1.104	1.105

*N* = 279. Gender was coded as 0 = male, 1 = female. Education was coded as 1 = below high school, 2 = high school, 3 = college, 4 = above college. Cronbach’s *α* are shown along the diagonal in bold italics. ^*^p < 0.05, ^**^p < 0.01.

### Hypotheses testing

**Figure 2:**
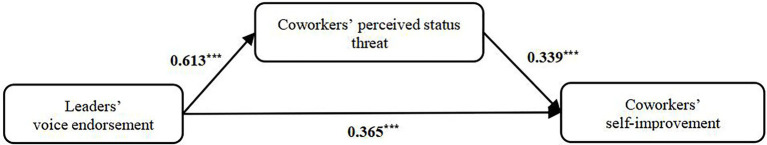
Results of path analysis. *N* = 279, ^**^p < 0.01, ^***^p < 0.001.

Path analysis was utilized to test hypotheses 1 and 2. As summarized in Figure 2, the positive effect of leaders’ voice endorsement on coworkers’ perceived status threat was significant after including the controls (*β* = 0.613, *p* < 0.001), and the positive effect of coworkers’ perceived status threat on self-improvement motivation was also significant (*β* = 0.339, *p* < 0.001). Consequently, H1 and H2 were supported.

All remaining hypotheses were tested using the PROCESS macro in SPSS 25.0 (Hayes and Scharkow, 2013) with a 5000-resample bootstrap method (Preacher et al., 2007). To examine hypothesis 3, PROCESS mode 4 was executed. As shown in Table 3, the result illustrated the significantly indirect effect of coworkers’ perceived status threat on the “leaders’ voice endorsement–self-improvement motivation” relationship (E.S. = 0.209, 95% bias-corrected CI = [0.148, 0.284]). Thus, H3 was supported.

**Table 3:** Coworkers’ perceived status threat as mediator in the relationship between leaders’ voice endorsement and coworkers’ self-improvement motivation.

Variables	Effect		Boot SE	Boot LL 95% CI	Boot UL 95% CI
Self-improvement motivation	Direct effect	0.363	0.060	0.244	0.482
	Indirect effect	0.209	0.034	0.148	0.284

All coefficients are unstandardized. SE, standard error; LL, lower level; UL, upper level; CI, confidence interval.

**Figure 3:**
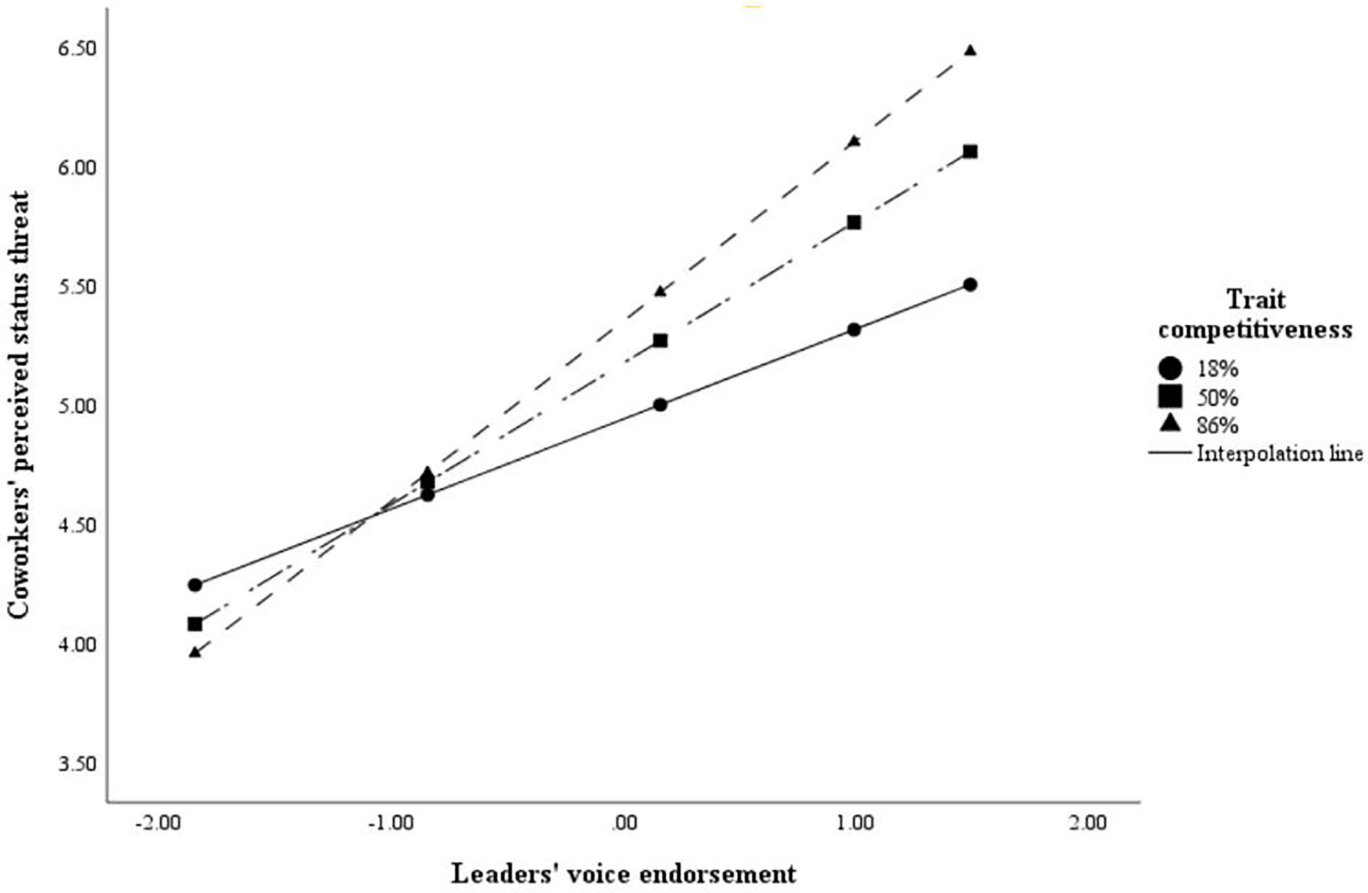
Interactive effect of leaders’ voice endorsement and trait competitiveness on coworkers’ perceived status threat.

PROCESS model 1 was executed to test H4. As shown in Table 4, it revealed that the interaction between leaders’ voice endorsement and trait competitiveness was significantly related to coworkers’ perceived status threat (E.S. = 0.135, SE = 0.043, 95% bias-corrected CI = [0.050, 0.220]). Following Hayes and Scharkow (2013), we plotted the interactions at 18%, 50%, and 86% percentiles of trait competitiveness. As shown in Figure 3, the effect of leaders’ voice endorsement on coworkers’ perceived status threat was stronger for coworkers with higher trait competitiveness. Thus, H4 was supported.

**Table 4:** Trait competitiveness as a moderator in the relationship between leaders’ voice endorsement and coworkers’ perceived status threat.

Variables	Effect	SE	Boot LL 95% CI	Boot UL 95% CI
Y: Coworkers’ perceived status threat				
Constant	5.038	0.382	4.287	5.790
M: Trait competitiveness	0.147	0.094	−0.037	0.332
X: leaders’ voice endorsement	0.593	0.088	0.419	0.767
Interaction: X × M	0.135	0.043	0.050	0.220

PROCESS model 58 was executed to test hypothesis 5. As shown in Table 5, the significant indirect effect of leaders’ voice endorsement on self-improvement motivation *via* coworkers’ perceived status threat was stronger when trait competitiveness was high (E.S. = 0.265, SE = 0.067, 95% bias-corrected CI = 0.146, 0.402]) than when it was low (E.S. = 0.132, SE = 0.036, 95% bias-corrected CI = [0.072, 0.213]). Thus, H5 was supported.

**Table 5:** Coworkers’ trait competitiveness as the moderator in conditional indirect effect model.

Variables	Trait competitiveness	Leaders’ voice endorsement → coworkers’ perceived status threat → self-improvement motivation			
		Effect	Boot SE	Boot LL95% CI	Boot UL95% CI
Self-improvement motivation	Low	0.132	0.036	0.072	0.213
	High	0.265	0.067	0.146	0.402

## Discussion

Based on self-evaluation maintenance theory, this study advanced and tested a model that explains how leaders’ voice endorsement motivates coworkers’ self-improvement motivation. Specifically, the research found that (1) leaders’ voice endorsement has a positive impact on coworkers’ perceived status threat; (2) coworkers’ perceived status threat has a positive effect on their self-improvement motivation; (3) coworkers’ perceived status threat mediates the relationship between leaders’ voice endorsement and coworkers’ self-improvement motivation; and (4) coworkers’ trait competitiveness strengthens the relationship between leaders’ voice endorsement and coworkers’ perceived status threat, and it also positively moderates the mediating effect of coworkers’ perceived status threat between leaders’ voice endorsement and coworkers’ self-improvement motivation.

The lapse in questionnaires that be submitted to analysis includes the following reasons. First, this study used a three-wave approach for data collection. The return rate for each wave is 88.2% (16 invalid leader questionnaires), 84.6% (65 invalid employee questionnaires), and 82.4% (63 invalid employee questionnaires). These rates are maintained at a good level of questionnaires return. However, since there is a certain interval between each wave, some employees are unable to fill out the questionnaire due to heavy workload, business trips, turnover, etc. In addition, if employees did not fill out the questionnaire in the previous wave, the study did not let these employees fill out the questionnaire in the next wave. Second, some questionnaires were excluded due to improper completion, such as the same answers to all items or with missing values. These reasons resulted in missing questionnaires submitted for analysis. Overall, however, the final 59.5% effective rate of the survey (279 sets of valid questionnaires) for this research remained within a good range. The good data collection process of this study provides the basis for data analysis.

### Theoretical implications

This study makes several theoretical contributions. First, the primary contribution of this work is that we shift attention from the antecedents of voice endorsement to the consequences of it. To our best knowledge, previous research on voice endorsement has largely focused on what factors facilitate endorsing voicers’ ideas. In addition, previously, research on voice endorsement has focused on the voicers themselves but ignored the potential effects of voice endorsement on other individuals or related personnel. Our research confirms the influence of the leaders’ voice endorsement on their coworkers, which fills the gap in the research field of voice endorsement. This study helps scholars gain a deep insight into the process of voice endorsement by taking the first step to proposing and revealing the positive effect of leaders’ voice endorsement on coworkers’ self-improvement motivation. It also directly responds to Morrison’s, (2011) call for future research to pay more attention to how employees’ voice affects their coworkers and their mutual relationships. Hence, our study provides new insights into voice research and enhances our understanding of the consequences of voice endorsement in the workplace.

Second, this study expands the outcome research on the perceived status threat by revealing the positive effect of coworkers’ perceived status threat on their self-improvement motivation. Most of the existing research on perceived status threat has focused on its negative effects. For example, the perceived status threat may lead to failure to change (Kellogg, 2012), intense conflicts in competitions (Bothner et al., 2007), and physiological stress responses (Scheepers and Ellemers, 2005). The results of our study found that when coworkers perceive status threats, they actively develop and enhance their capabilities due to the deviation of their situation. This finding not only enriches the outcomes of the role of perceived status threat but also provides new ideas for future related research.

Finally, this study validates the moderating role of trait competitiveness and examines its boundary effect on voice endorsement. In the complex arena of organizations, individual personality traits are extremely important in explaining their workplace behaviors. Coworkers with high trait competitiveness are more sensitive to status gains and losses and are more willing to participate in the competition. The empirical results of this research demonstrated that coworkers’ trait competitiveness significantly enhances the positive connection between leaders’ voice endorsement and coworkers’ perceived status threat. It clarifies the moderating effect of trait competitiveness and helps to deepen the understanding of the process by which leaders’ voice endorsement affects the psychology of their employees.

### Practical implications

This study provides several practical implications. First, this study found that leaders’ voice endorsement could lead to coworkers’ stress response, that is, perception of status threat. To reduce the possible negative effects of voice endorsement, managers should create a relaxed perception of the consequences of voice in the organization, encourage employees to express their ideas, share information, and build high-quality communication channels. Employees should also pay attention to the skills and strategies when making suggestions to leaders. For example, employees should consider the status threat impact of their suggestions on coworkers and try to bring benefits to coworkers as well. By choosing the right ways and time to make work-related suggestions to leaders, employees can effectively reduce coworkers’ worries and suspicions. Through the collaboration between the organization and individuals, the positive effects of voice endorsement can be amplified and its possible negative effects can be avoided.

Second, this research found that employees’ self-evaluation maintenance is an important factor in their perception of status threat. The self-evaluation maintenance model suggests that comparisons with better-performing employees can lead to self-perceived threats. The evaluation bias produced by self-evaluation can lead to an imbalance in self-positioning. Therefore, for the organization, an open, transparent, fair, and just evaluation system should be established to give employees objective evaluation results. For employees, they should clarify their strengths and weaknesses so that they can set their mindset.

Third, this study found that employees’ perceived status threat has a positive effect on their self-development. Although some research has suggested that status threat mainly brings negative outcomes, this study originally found that it also has a positive effect on employees. The imposition of some status threat to employees can increase their desire for self-improvement, which, in turn, facilitates the development of their capabilities. Therefore, the organization should reasonably build a competitive atmosphere and promote healthy competition among employees, to effectively utilize their perception of status threat and enhance their work ability.

### Limitations and future research

This study may have several potential limitations. First, this research only explored the boundary effects of personal traits (i.e., trait competitiveness) without considering other factors. Both organizational and cultural factors may also be used to study moderating effects. Therefore, future research can continue to explore the moderating role of different organizational and cultural factors on the relationship between voice endorsement and perceived status threat. Second, although this study has validated the mediating role of perceived status threat between voice endorsement and self-improvement motivation from the perspective of self-evaluation, the mechanisms by which employees’ voice endorsement influences the psychological and behavioral outcomes of coworkers may be diverse. Future research should explore the effects of voice endorsement on individuals and teams from different theoretical perspectives. Third, the sample of this study came from Chinese manufacturing companies, which inhibited the external validity of the study despite enhancing the internal validity. Therefore, future research should use samples from multiple companies, industries, and regions for data surveys to further test the findings of this study.

## Data availability statement

The raw data supporting the conclusions of this article will be made available by the authors, without undue reservation.

## Ethics statement

This study was carried out in accordance with the recommendations of ethical guidelines of the Ethical Review Board of Beijing Wuzi University. The protocol was approved by the Ethical Review Board of Beijing Wuzi University. The patients/participants provided their written informed consent to participate in this study in accordance with the Declaration of Helsinki. Written informed consent was obtained from the individual(s) for the publication of any potentially identifiable images or data included in this article.

## Author contributions

PL made great contributions to supervision and the analysis of data for the study, and revised it critically for important intellectual content. DL made contributions to the conception of the study and wrote the manuscript. XZ contributed to the acquisition of data for the study. All authors contributed to the article and approved the submitted version.

## Conflict of interest

The authors declare that the research was conducted in the absence of any commercial or financial relationships that could be construed as a potential conflict of interest.

## Publisher’s note

All claims expressed in this article are solely those of the authors and do not necessarily represent those of their affiliated organizations, or those of the publisher, the editors and the reviewers. Any product that may be evaluated in this article, or claim that may be made by its manufacturer, is not guaranteed or endorsed by the publisher.
